# Differential control of two forms of glutamate release by group III metabotropic glutamate receptors at rat entorhinal synapses

**DOI:** 10.1016/j.neuroscience.2007.06.002

**Published:** 2007-08-10

**Authors:** G.L. Woodhall, G. Ayman, R.S.G. Jones

**Affiliations:** a Physiology and Pharmacology, School of Life and Health Sciences, Aston University, Birmingham B4 7ET, UK; b Department of Pharmacy and Pharmacology, University of Bath, Claverton Down, Bath BA2 7AY, UK

**Keywords:** presynaptic metabotropic receptors, entorhinal cortex, glutamate release, AC, adenylyl cyclase, ACPT-1 (1S, 3R,4S)-1-aminocyclopentane-1,2,4-tricarboxylic acid, ACSF, artificial cerebrospinal fluid, AgTx, agatoxin IVA, AP, action potential, CPPG, (RS)-cyclopropyl-4-phosphonophenylglycine, CTx, ω-conotoxin GVIA, EC, entorhinal cortex, eEPSC, evoked excitatory postsynaptic current, IEI, inter-event interval, KS, Kolmogorov-Smirnoff, mEPSC, miniature excitatory postsynaptic current, mGluR, metabotropic glutamate receptor, mIPSC, miniature inhibitory postsynaptic current, NMDA, *N*-methyl-d-aspartate, PKA, protein kinase A, RRP, readily releasable pool, sEPSC, spontaneous excitatory postsynaptic current, SNx, SNX-482, SQ22536, 9-tetrahydro-2-furanyl)-9H-purin-6-amine, TTX, tetrodotoxin, VGCC, voltage-gated calcium channel

## Abstract

Neurotransmitter release at CNS synapses occurs via both action potential-dependent and independent mechanisms, and it has generally been accepted that these two forms of release are regulated in parallel. We examined the effects of activation of group III metabotropic glutamate receptors (mGluRs) on stimulus-evoked and spontaneous glutamate release onto entorhinal cortical neurones in rats, and found a differential regulation of action potential-dependent and independent forms of release. Activation of presynaptic mGluRs depressed the amplitude of stimulus-evoked excitatory postsynaptic currents, but concurrently enhanced the frequency of spontaneous excitatory currents. Moreover, these differential effects on glutamate release were mediated by pharmacologically separable mechanisms. Application of the specific activator of adenylyl cyclase, forskolin, mimicked the effect of mGluR activation on spontaneous, but not evoked release, and inhibition of adenylyl cyclase with 9-tetrahydro-2-furanyl)-9H-purin-6-amine (SQ22536) blocked mGluR-mediated enhancement of spontaneous release, but not depression of evoked release. Occlusion studies with calcium channel blockers suggested that the group III mGluRs might depress evoked release through inhibition of both N and P/Q, but not R-type calcium channels. We suggest that the concurrent depression of action potential-evoked, and enhancement of action potential-independent glutamate release operate through discrete second messenger/effector systems at excitatory entorhinal terminals in rat brain.

Transmitter release at central synapses has two components, that driven by action potentials (APs) invading the presynaptic terminal, and an AP-independent component which reflects quantal release. AP-dependent release is often multi-quantal, and depends on calcium entry through voltage-gated calcium channels (VGCCs; see Spafford and Zamponi, 2003, for review). Toxins that block VGCCs depress AP-dependent release (e.g. see Yeager et al., 1987; Llinas et al., 1989). In contrast, AP-independent (‘miniature’) neurotransmitter release reflects stochastic release of transmitter quanta from individual vesicles, and can occur at basal calcium levels when APs are blocked (e.g. Otis et al., 1991). In addition to these studies, Sara et al. (2005) have demonstrated that, in hippocampal cultures, the pool of vesicles underlying miniature release may be separate from that underlying evoked release (but see Groemer and Klingauf, 2007), and it has been suggested that the presynaptic protein, synaptobrevin, may regulate vesicle transfer between separate vesicle pools (Zucker, 2005).

Previous studies have suggested that AP-dependent and independent neurotransmitter release are regulated in parallel (Prange and Murphy, 1999; Dietrich et al., 2002), but there is also evidence to suggest that they may be differentially regulated. For example, application of noradrenaline to cultured hippocampal neurones decreases the amplitude of evoked excitatory responses, without alteration of amplitude or frequency of miniature excitatory postsynaptic currents (mEPSCs; Scanziani et al., 1993). In cerebellar stellate neurones, noradrenaline increases the frequency but not amplitude of miniature inhibitory postsynaptic currents (mIPSCs), while concurrently reducing the amplitude of evoked inhibitory postsynaptic currents (Llano and Gerschenfeld, 1993; Kondo and Marty, 1998). At parallel fiber synapses onto cerebellar Purkinje cells, activation of metabotropic glutamate receptors (mGluRs), probably mGluR1, increases the frequency of spontaneous excitatory postsynaptic currents (sEPSCs) but concurrently reduces the amplitude of evoked excitatory postsynaptic currents (eEPSCs; Levenes et al., 2001). Differential regulation of evoked and spontaneous glycine release at spinal cord synapses has also been demonstrated (Katsurabayashi et al., 2004). Finally, it has been consistently shown that VGCC blockers, which abolish evoked release, have little effect on the frequency or amplitude of miniature currents in many preparations (e.g. del Castillo and Katz, 1954; Katz and Miledi, 1968; Scanziani et al., 1992, 1995; c.f. Hori et al., 1999).

At synapses on layer V neurones in the rat entorhinal cortex (EC), we have previously shown that group III mGluRs enhanced the spontaneous release of glutamate (Evans et al., 2000a). This unusual enhancement occurred via a direct modulation of glutamate release involving protein kinase A (PKA) and PKC (Evans et al., 2001), and was evidenced by an increase in the frequency of sEPSCs. In the population data this was accompanied overall by a small increase in mean amplitude, but in some individual neurones, there was an increase in frequency accompanied by a clear decrease in mean amplitude with group III agonists, reflecting a loss of larger amplitude events. However, when we recorded mEPSCs, the increased frequency still occurred, but with no change in amplitude distribution. The change in mEPSCs would be unlikely ascribed to effects on VGCCs as mGluRs are considered to reduce the activation of these channels (Glaum and Miller, 1995; Takahashi et al., 1996). This led us to consider the possibility that mGluR activation could have differential effects on AP-independent and multi-quantal, AP-dependent release, thus differentially modulating the two forms of release. In the present study we demonstrate that increased spontaneous release occurs concurrently with a decrease in the amplitude of eEPSCs, and we have attempted to clarify the mechanism responsible for these effects. Some of these results have been presented in abstracts (Evans et al., 2000b; Jones et al., 2004).

## Experimental procedures

Combined EC–hippocampal slices were prepared from young male Wistar rats (50–110 g) as previously described (Jones and Heinemann, 1988). All experiments were performed in accordance with the U.K. Animals (Scientific Procedures) Act 1986, European Communities Council Directive 1986 (86/609/EEC) and the Bath, Bristol and Aston University ethical review documents. Every effort was made to minimize the number of animals used and their suffering. Rats were anesthetized with an i.m. injection of ketamine (120 mg/kg) plus xylazine (8 mg/kg) and decapitated. The brain was rapidly removed and immersed in oxygenated artificial cerebrospinal fluid (ACSF) chilled to 4 °C. Slices (450 μm) were cut using a Vibroslice (Campden Instruments, Loughborough, UK), and stored in ACSF continuously bubbled with 95% O_2_/5% CO_2_, at room temperature. Following a recovery period of at least 1 h, individual slices were transferred to a recording chamber mounted on the stage of an Olympus (Tokyo, Japan; BX50WI) or Zeiss Axioskop FS (Carl Zeiss, Oberkochen, Germany) upright microscope. The chamber was continuously perfused with oxygenated ACSF at 30–32 °C, at a flow rate of approximately 2 ml/min. The ACSF contained the following (in mM): NaCl (126), KCl (3.25), NaH_2_PO_4_ (1.25), NaHCO_3_ (24), MgSO_4_ (2), CaCl_2_ (2.5), and d-glucose (10). The solution was continuously bubbled with 95% O_2_/5% CO_2_ to maintain a pH of 7.4. Neurones were visualized using differential interference contrast optics and an infrared video camera.

Patch clamp electrodes were pulled from borosilicate glass (1.2 mm OD, 0.69 ID; Harvard Apparatus, Holliston, MA, USA) and had open tip resistances of 4–5 MΩ. They were filled with a solution containing the following (in mM): Cs-methanesulphonate (130), Hepes (10), QX-314 (5), EGTA (0.5), NaCl (1), CaCl_2_ (0.34), ATP (4), GTP (0.4). The solution was adjusted to 290 mOsmol with sucrose and to pH 7.4 with CsOH. Whole-cell voltage clamp recordings were made from neurones in layer V of the medial division of the EC (mEC), using an Axopatch 200B amplifier (Molecular Devices, Sunnyvale, CA, USA). The holding potential in all cases was −60 mV. Under these experimental conditions, layer V neurones exhibit sEPSCs mediated by glutamate acting primarily at AMPA receptors (see Berretta and Jones, 1996a). Pure *N*-methyl-d-aspartate (NMDA)-receptor-mediated sEPSCs also occur at a much lower frequency (Berretta and Jones, 1996a), but in the present study no attempt was made to discriminate the effects of the drugs on events mediated by the two receptors.

eEPSCs were elicited by electrical stimulation (bipolar pulses, 10–100 mV, 0.02 ms duration) via a bipolar tungsten electrode placed on the surface of the slice in layer V of the lateral EC. Evoked responses in the mEC can vary greatly in peak amplitude, and from trial to trial, so we adjusted stimulation intensity to always give a sub-maximal (approx. 2/3 maximum amplitude) response, and used of trains of eEPSCs to mitigate single trial variability. For paired-pulse and high frequency trains, an inter-pulse interval of 50 ms was used. For all protocols, stimulation was applied once every 30 s for the duration of the experiment. sEPSCs (consisting of both AP-dependent and independent events) were recorded continuously between eEPSCs. Access resistance was monitored at regular intervals and cells were rejected if this parameter changed by more than ±15%.

Data were recorded directly to computer hard disk using AxoScope software (Molecular Devices). MiniAnalysis (Synaptosoft, Decatur, GA, USA) was used for analysis of both eEPSCs and sEPSCs off-line. For each cell, under each condition, 10 eEPSCs were averaged and peak eEPSC amplitude determined. A paired Student’s *t*-test was used for comparison of eEPSC amplitudes. sEPSCs were detected automatically using a threshold-crossing algorithm, and their frequency and amplitude analyzed. Two hundred sEPSCs were sampled during a continuous recording period for each neurone under each condition. The non-parametric Kolmogorov-Smirnoff (KS) test was used to assess the significance of shifts in cumulative probability distributions of inter-event interval (IEI) (Van der Kloot, 1991). Differences between drug and control situations in studies of eEPSCs were assessed by means a one-way ANOVA. All error values stated in the text refer to the S.E.M.

All salts used in preparation of ACSF were Analar grade and purchased from Merck/BDH (Poole, UK). (1S,3R,4S)-1-Aminocyclopentane-1,2,4-tricarboxylic acid (ACPT-1), forskolin and (RS)-cyclopropyl-4-phosphonophenylglycine (CPPG) were obtained from Tocris Cookson (Bristol, UK). 9-Tetrahydro-2-furanyl)-9H-purin-6-amine (SQ22536) was purchased from RBI (Manchester, UK), tetrodotoxin (TTX) and SNX-482 (SNx) from Alamone Laboratories (Jerusalem, Israel), and ω-conotoxin GVIA (CTx) and agatoxin IVA (AgTx) from Sigma (Gillingham, UK).

## Results

### Presynaptic mGluR activation enhances glutamate release

**Fig. 1:**
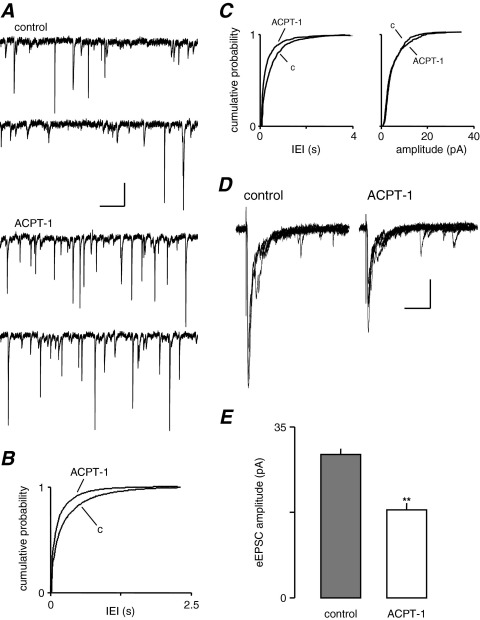
ACPT-1 differentially modulates sEPSCs and eEPSCs. (A) Voltage clamp recording from a layer V neurone illustrating the increase in sEPSCs elicited by ACPT-1. (B) Cumulative probability analysis of interevent interval of sEPSCs in pooled data (*n*=10). The shift to the left of the distribution in ACPT-1 reflects the increase in frequency compared with control (c). (C) Pooled data of cumulative probability analysis of mEPSCs showing the persistence of the increase in frequency with no significant change in amplitude (*n*=7). (D) eEPSCs (five events superimposed) recorded in the same neurone shown in A, illustrating the concurrent reduction of AP-dependent-release. (E) Summary data for eEPSC changes in the same set of neurones as in B, showing the consistent reduction in eEPSCs concurrent with the increased frequency of sEPSCs. Scale bars=15 pA, 500 ms (A); 30 pA, 30 ms (D).

We have previously demonstrated that application of the group III mGluR agonist, L-AP4, facilitated spontaneous and AP-independent ‘miniature’ glutamate release (recorded in the presence of TTX) onto layer V neurones (Evans et al., 2000a). Here, we examined the effect of the potent agonist of group III mGluR, ACPT-1 (20 μM; Acher et al., 1997) on sEPSCs. As Fig. 1A shows, bath application of ACPT-1 increased the frequency of sEPSCs reflected by the change in the distribution toward shorter IEIs (*P*<0.05, KS; Fig. 1B), with a mean IEI of 256±3 ms under control conditions and 151±3 ms in the presence of ACPT-1. We also made recordings in the presence of TTX (1.25 μM; *n*=7). Again, ACPT-1 caused a shift in the distribution of IEI (Fig. 1C) toward shorter intervals, reflecting a substantial increase in the frequency of AP-independent events (*P*<0.05, KS). The mean IEI of mEPSCs was decreased from 666±17 ms to 301±3 ms in the presence of ACPT-1, reflecting a doubling of the frequency. Pooled data from the same seven neurones (Fig. 1D) showed that the amplitude distribution of mEPSCs was unaffected by APCT-1 (*P*>0.5, KS; mean amplitude 10.1±0.2 pA in TTX and 10.2±0.2 in ACPT-1). The decreased IEI without change in the amplitude of mEPSCs indicated that ACPT-1 increased AP-independent release of glutamate through a presynaptic action, consistent with our previous studies (Evans et al., 2000a, 2001). Kinetic analyses revealed no differences in the distribution of rise and decay times of mEPSCs (*P*>0.5 in both cases, KS; data not shown), supporting the conclusion that the effects of the mGluR agonist on IEI were due to presynaptic modulation of glutamate release.

### mGluR activation reduces eEPSC amplitude

Having established that the effects of ACPT-1 were mediated by a presynaptic receptor, we next examined the effect of mGluR activation on the evoked component of glutamate release under conditions in which network activity was intact, and both sEPSCs and eEPSCs could be monitored. In recordings from 10 layer V neurones, the mean frequency of sEPSCs was increased during bath application of ACPT-1 (20 μM ACPT-1), as described above and previously reported (Evans et al., 2000a). Concurrently, however, the mean amplitude of eEPSCs was reduced from 29.4±1.2 pA to 18.0±1.4 pA (*P*<0.05, paired *t*-test). Fig. 1D shows voltage clamp recordings of eEPSCs from one neurone (from the same cell illustrated in Fig. 1A). Summary data for the 10 neurones are illustrated in Fig. 1E. Since the majority of sEPSCs in layer V are TTX-insensitive, miniature events, and ACPT-1 increases the frequency of mEPSCs (Fig. 1C and Evans et al., 2000a), these experiments clearly indicate a differential modulation by mGluR of evoked and spontaneous release at these synapses.

### mGluR blockade reveals tonic effects on glutamate release

**Fig. 2:**
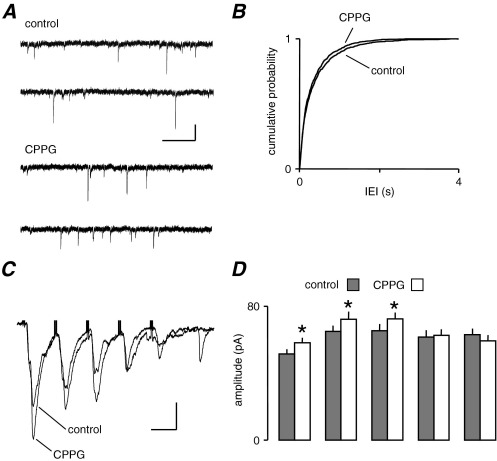
Blockade of mGluRs with CPPG reveals tonic effects on eEPSCs but not sEPSCs. (A) sEPSCs recorded from a layer V neurone showing little effect of CPPG (2 μM). (B) The cumulative probability plot of pooled data from eight neurones shows a slight shift toward shorter intervals but this was not significant, indicating no change in frequency. (C) The traces are averaged records (*n*=6) of eEPSCs evoked at 20 Hz. CPPG enhanced the amplitude of early events in the train. (D) The bar chart shows pooled data from eight cells in which mean eEPSC amplitude was measured during the train. The amplitude of the first three eEPSCs in a train was significantly enhanced, but the later events were unaffected. Scale bars=15 pA, 100 ms (A); 20 pA, 30 ms (C).

Given that mGluR activation enhanced spontaneous glutamate release and depressed evoked release, it could be expected that mGluR antagonists might show opposite effects. We applied the group III mGluR antagonist, CPPG (2 μM), while recording both sEPSCs and eEPSCs. Fig. 2A shows that a tonic effect of mGluRs on sEPSC frequency is unlikely, since there was no obvious or dramatic change in sEPSC frequency. The pooled data (Fig. 2B) did show a small leftward shift in IEI distribution toward shorter intervals in CPPG but this did not reach significance (*P*>0.05, KS). Similar results were obtained with CPPG at 10 μM (not shown). In contrast, we did observe a robust change in eEPSC amplitude in response to CPPG application. The mean peak amplitude of eEPSCs increased from 48.2±4.5 pA to 57.4±3.1 pA (*P*<0.05, paired *t*-test; *n*=8). Fig. 2C shows a train of five events recorded at 20 Hz in one neurone. With such a train we usually observed an initial facilitation followed by depression of eEPSCs, or occasionally, as in this case, a simple depression. Clearly, in the presence of CPPG there was an enhancement of eEPSCs, particularly of the first three events. In pooled data (Fig. 2D, *n*=8), the overall profile was initial facilitation, which then plateaued or slightly depressed. CPPG did not alter the profile, but consistently and significantly increased the size of events early in the train. Interestingly, the amplitude of the first event in a train was always facilitated by CPPG, suggesting a constitutive effect via group III mGluRs on AP-dependent release of glutamate at these synapses. In the following experiments we attempted to determine the second messenger events underlying the differential control of glutamate release responsible for sEPSCs and eEPSCs.

### Baclofen does not block the effects of ACPT-1

Previous experiments have demonstrated that activation of presynaptic GABA_B_ receptors (GABA_B_R) inhibits both AP-dependent and independent GABA release in layer V of the EC, although the intracellular mechanisms have yet to be elucidated (Bailey et al., 2004). GABA release in layer V is also decreased by group III mGluR activation (Woodhall et al., 2001a). It is possible that GABA_B_R and group III mGluRs may couple to common intracellular components, and this may also apply to excitatory synapses and glutamate release. Thus, it was of interest to determine whether the activation of GABA_B_Rs could occlude the effect of ACPT-1 on evoked and spontaneous glutamate release. Again, we utilized trains of stimuli at 20 Hz and measured the peak amplitude of all events within the train to reduce the influence of intrinsic variability of eEPSCs in these and subsequent occlusion experiments.

**Fig. 3:**
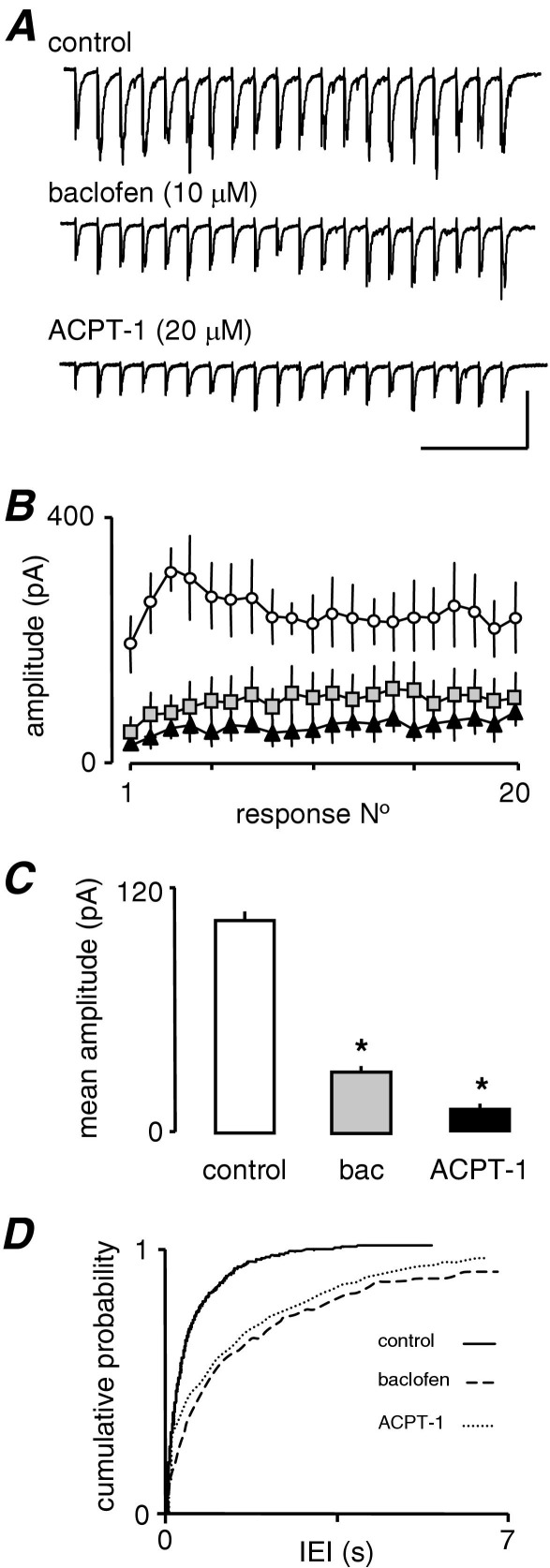
The effects of ACPT-1 are not occluded by GABA_B_ receptor activation. (A) Single voltage clamp recordings of eEPSCs evoked in response to a 20 Hz train of 20 stimuli in the presence of the GABA_B_R agonist baclofen show a pronounced depression across the train. Subsequently, ACPT-1 in the continued presence of baclofen, elicited a further depression. (B) Pooled mean eEPSC amplitude (*n*=7) for each event in a 20 Hz train (C) Pooled analysis of mean eEPSC amplitude from all events in the train from seven neurones. The substantial reduction by baclofen does not occlude a further significant depression by ACPT-1. (D) Cumulative probability analysis of sEPSCs in the seven neurones. A dramatic reduction in frequency by baclofen is partially reversed by ACPT-1. Scale bars=150 pA, 225 ms (A).

Fig. 3A shows trains of eEPSCs evoked at 20 Hz for 1 s in one neurone and reveals that the GABA_B_R agonist, baclofen (10 μM), substantially depressed the amplitude of all events within the train. The graphs in Fig. 3B show averaged events from seven neurones and illustrate the depression of eEPSC amplitude of all 20 eEPSCs. Baclofen depressed all events in the train significantly (*P*<0.05, *t*-test). It is clear that the most pronounced effect occurred in the first half of the train reducing the largest events to the extent that an initial facilitation–depression profile of eEPSCs was converted to a gradual facilitation alone (Fig. 3B). It is also abundantly clear from Fig. 3A and 3B that the addition of ACPT-1 in addition to baclofen caused a further depression eEPSCs across the train, despite the large reduction already elicited by baclofen. ACPT-1 depressed the events considerably when expressed as a percentage of the amplitude of the events following baclofen application (approximately 40%). The change in the overall amplitude of eEPSCs in the train (averaged across all 20 events) was highly significant in both cases. As means of quantifying the overall effect of the drugs we averaged amplitudes of eEPSCs across the whole train. In the example shown, baclofen decreased the mean amplitude from 249.0±12.5 pA to 101.7±7.1 pA, and ACPT-1 further decreased mean amplitude, to 60.6±5.6 pA (*P*<0.001, ANOVA), which equates to a percentage reduction similar to that determined previously with ACPT-1 alone. Fig. 3C summarizes the mean results of analysis in four neurones.

In addition, ACPT-1 appeared to remain effective at increasing the frequency of sEPSCs in the presence of baclofen. Fig. 3D shows a pronounced rightward shift in IEI distribution in the presence of baclofen (same neurones as in Fig. 3C). This shift is partially reversed by ACPT-1, although the low frequency of events in baclofen means that this effect is not dramatic. Thus, these data indicate that ACPT-1, and hence group III mGluR activation, is still able to influence evoked and AP-independent glutamate release under conditions in which the GABA_B_Rs are strongly activated. The lack of occlusion suggests that group III mGluRs and GABA_B_Rs show little convergence at the level of G-proteins or second messengers, and also that GABA_B_Rs depress both eEPSC amplitude and sEPSC frequency. We next investigated the second messenger systems underlying the differential modulation of eEPSC amplitude and sEPSC frequency induced by the group III mGluRs.

### Forskolin enhances sEPSC frequency and eEPSC amplitude

**Fig. 4:**
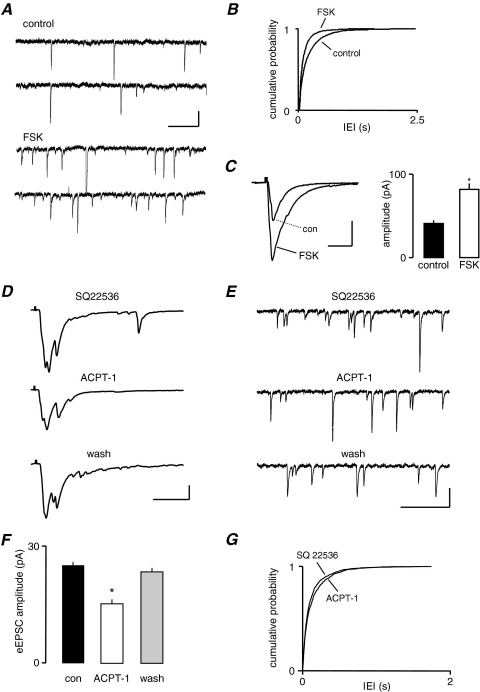
The depression of eEPSC amplitude by ACPT-1 is not mediated by PKA. (A) sEPSCs recorded before and during application of forskolin (FSK, 20 μM) showing a clear increase in frequency. (B) This is confirmed by the cumulative probability analysis for pooled sEPSCs from seven neurones. (C) Averaged eEPSC responses (*n*=10) from the same cell as in A, showing that FSK increases eEPSC amplitude, and this is clearly shown by the pooled amplitude data in the bar chart, from the same seven neurones as in B. (D) Individual eEPSCs recorded in the presence of the specific AC inhibitor SQ22536 (50 μM) alone, and with the subsequent addition of ACPT-1 (20 μM). The agonist induced a similar depression of the eEPSC amplitude to that seen without the AC inhibitor. (E) sEPSCs recorded from the same neurone as in D showing the lack of effect of ACPT-1 on spontaneous release during inhibition of AC. (F) The bar graph summarizes changes in eEPSC amplitude with ACPT-1 during application of SQ22536 in seven neurones. (G) The cumulative probability plot shows pooled data for IEI in the same seven neurones as in F. Scale bars=10 pA, 60 ms (A); 60 pA, 20 ms (C, D); 20 pA, 20 ms (E).

We previously demonstrated that group III mGluR activation enhanced sEPSC frequency by a direct modulation of glutamate release via PKA (Evans et al., 2001). PKA has also been shown to regulate AP-dependent transmitter release via modulation of presynaptic calcium channels (Ghirardi et al., 1992; Hell et al., 1995; Tong et al., 1996; Katsurabayashi et al., 2004). To investigate whether PKA is also involved in ACPT-1 mediated suppression of eEPSCs we examined the effect of the specific adenylyl cyclase (AC) activator, forskolin. In seven neurones, the mean IEI of sEPSCs was 476±20 ms under control conditions and this decreased to 222±15 ms during bath application of forskolin. An example is shown in Fig. 4A and pooled data from the seven neurones (Fig. 4B) show a clear decrease in IEI (*P*<0.01, KS). However, in the same neurones, the mean amplitude of concurrently recorded eEPSCs was increased from 49.2±4.4 pA to 98.6±8.3 pA in the presence of forskolin (*P*<0.01). Fig. 4C shows averaged eEPSCs recorded in one neurone and pooled data for peak amplitude changes in all neurones tested. Because forskolin caused a clear increase in the mean amplitude of eEPSCs it is unlikely that PKA mediates the depression of eEPSC amplitude following mGluR activation. The inactive forskolin analog 2,4-dideoxyforskolin had no effect on eEPSCs, suggesting that we were not observing non-specific actions of the PKA activator (Evans et al., 2001).

### The effect of ACPT-1 persists in the presence of SQ22536

In order to confirm that the depression of eEPSC amplitude by ACPT-1 was not due to activation of the AC/PKA pathway, we preincubated slices in ACSF containing the specific AC inhibitor, SQ22536 (50 μM; Fabbri et al., 1991), and continued its application throughout the subsequent period of recording. Fig. 4D and 4E show sample records from such an experiment. In the presence of SQ22536, bath application of ACPT-1 reduced the amplitude of eEPSCs, similar to that observed under control conditions (Fig. 4D). Mean eEPSC amplitude was 31.2±1.2 pA under control conditions and 19.3±0.8 pA during application of ACPT-1 (bar graph Fig. 4F; *P*<0.01, *n*=7). However, SQ22536 did prevent the increase in sEPSC frequency elicited by ACPT-1. Fig. 4E shows voltage clamp records illustrating the lack of effect of ACPT-1 in the presence of SQ22536. Pooled data from the seven neurones show the overlapping cumulative probability distributions of IEI reinforce this observation (Fig. 4G). These data clearly indicate that while the facilitatory effect on sEPSCs is mediated via a positive coupling to the AC/PKA pathway, the reduction in eEPSC amplitude involves a different messenger–effector pathway.

### ACPT-1 depresses paired-pulse facilitation

**Fig. 5:**
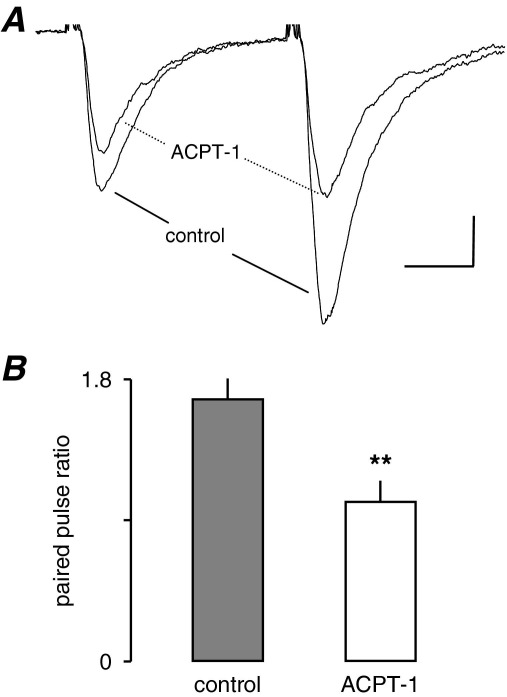
ACPT-1 decreases the paired-pulse ratio. (A) Averaged eEPSCs (*n*=6) recorded from a layer V neurone before and after application of ACPT-1. (B) Pooled data from seven neurones showing the significant reduction in paired-pulse facilitation in ACPT-1. Scale bar=30 pA, 12 ms (A).

The lack of depression of eEPSC amplitude indicated that second messenger system(s) underlying enhancement of sEPSCs was different to that underlying depression of AP-dependent eEPSCs. Since it is well established that AP-dependent release involves calcium entry through voltage-gated channels, we tested the effect of ACPT-1 on paired-pulse facilitation, a form of short-term plasticity that involves an increase in an evoked response as a result of a preceding conditioning stimulus. It is specific to the set of afferent inputs excited by the first stimulus and is widely believed to depend on residual Ca^2+^ in the presynaptic terminals (e.g. Hess and Kuhnt, 1992). Paired-pulse stimulation was examined in seven neurones (Fig. 5) using an inter-pulse interval of 50 ms. The effect of ACPT-1 was to decrease both eEPSCs although there was clearly a greater effect on the test response (Fig. 5A). In seven neurones tested the mean paired-pulse ratio was 1.7±0.1 and in the presence of ACPT-1 this was decreased to 1.1±0.2. (*P*<0.01, *n*=7; Fig. 5B). These data suggest that ACPT-1 modifies eEPSCs by a direct action at the presynaptic terminal suggesting that group III mGluR activation depresses eEPSCs by reducing Ca^2+^-entry, a possibility we investigated next.

### Blockade of VGCCs alters mGluR-mediated depression of eEPSCs

**Fig. 6:**
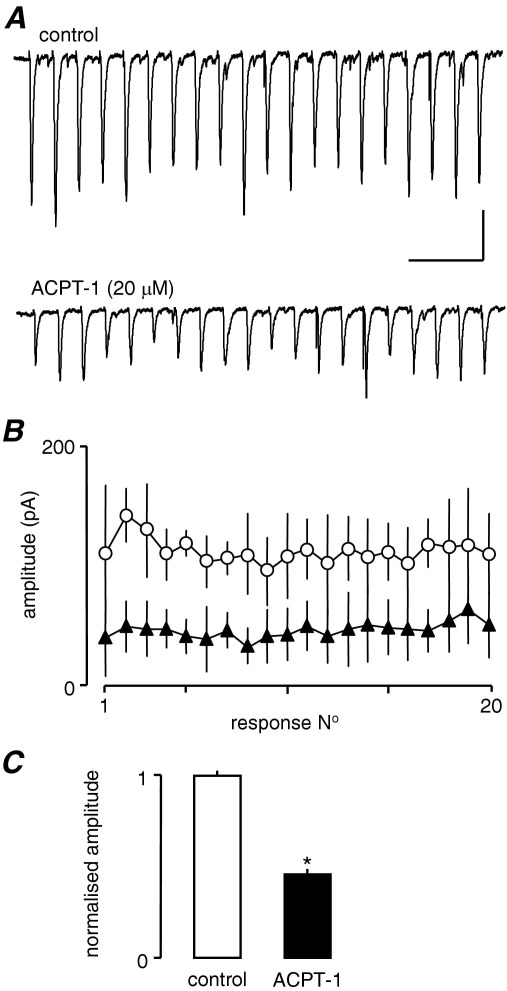
ACPT-1 depresses eEPSCs across a 20 Hz train. (A) Single recordings show an approximately 50% depression of eEPSCs across the train and a flattening of the facilitation–depression profile seen in most neurones. (B) Pooled data from four trains each in three neurones. (C) Averaged normalized fractional amplitudes of all events in the four trains from the three neurones. Scale bar=100 pA, 250 ms (A).

Previous studies indicate that the depression of glutamatergic transmission by mGluRs derives from inhibition of VGCCs in the presynaptic terminal (Glaum and Miller, 1995; Takahashi et al., 1996; Millan et al., 2002, but cf Rusakov et al., 2004). Of the functionally categorized subtypes of VGCCs, the bulk of evidence suggests that glutamate release underlying fast synaptic transmission is predominantly mediated by P/Q and/or N-type channels (e.g. Wu and Saggau, 1994; Reid et al., 2003; Wu et al., 1999; Qian and Noebels, 2001), although there is increasing evidence that R-type channels may also contribute (Wu et al., 1998, 1999; Krieger et al., 1999; Gasparini et al., 2001). We made use of specific inhibitors to determine whether a reduction in presynaptic voltage-gated Ca^2+^-influx may underlie the ACPT-1-mediated suppression of AP-dependent glutamate release. CTx (1 μM) was used to block N-type channels, and AgTx (200 nM) and SNx (1 μM) were used to block P/Q and R-type channels, respectively. We conducted our toxin experiments in the presence of ascorbic acid (450 μM), since this has been reported to greatly reduce the concentrations of channel toxins required for effective blockade (Casali et al., 1997). Ascorbate alone had no effect on eEPSCs (not shown). As discussed above, eEPSCs in layer V show considerable fluctuation in amplitude from trial-to-trial so, again, we employed stimulus trains (20 Hz, 1 s) in this series of experiments. eEPSC amplitude remained variable throughout the trains, but averaging eEPSCs across trains provided a more consistent picture of the synaptic events, facilitating comparisons between experiments. As before, repetitive stimulation induced variable profiles of evoked responses, with the most common being an initial facilitation of followed by a subsequent decline back to control levels, or beyond this to an overt depression. Fig. 6A shows the effect of ACPT alone (in the presence of ascorbate) in one neurone and clearly shows the depression of all events in the train. Averaged data (four trains in each of three neurones) are shown in Fig. 6B. The bar graphs in Fig. 6C show the mean fractional amplitude of events across the trains in the three neurones, normalized to the mean control amplitude in each neurone. ACPT alone produced around a 50% reduction in overall amplitude of eEPSCs.

**Fig. 7:**
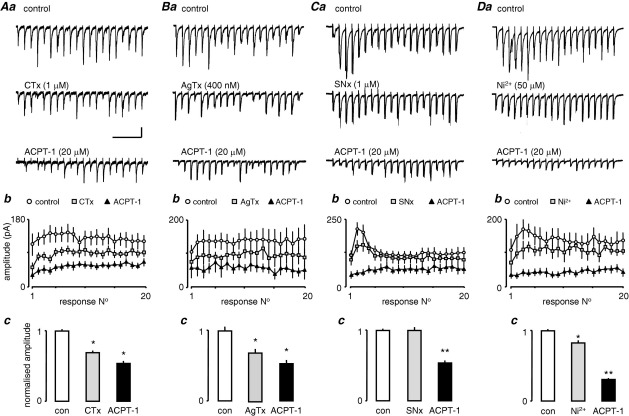
ACPT-1 reduces eEPSC amplitude after blockade of VGCCs. Details as for Fig. 6. (Aa) eEPSCs evoked at 20 Hz, 1 s train during perfusion with CTx and with the subsequent addition of ACPT-1 (in the continued presence of toxin). (Ab) Summary data in 17 neurones. During perfusion with CTx eEPSCs were reduced across the train, but still reduced further by ACPT-1 to around the same overall extent as with ACPT-1 alone (Fig. 6). (B) AgTx, had very similar effects. (C) In the case of SNx, the reduction in eEPSC amplitude was largely confined to the early facilitated responses (Ca, b) with no overall depression across the whole train (Cc). ACPT-1 was just as effective as when applied alone (Fig. 6). (D) Ni^2+^ weakly expressed events across the whole train (Da, b) and subsequent addition of ACPT-1 had a more pronounced effect than with ACPT-1 alone (Dc, cf Fig. 6C; *n*=17 neurones in CTx; *n*=7 in AgTx; *n*=6 in SNx and Ni^2+^). Scale bar=40 pA, 250 ms for all recordings.

Fig. 7 shows the effects of prior perfusion with VGCC blockers. After a control period of recording, the blockers were applied until maximal effects on eEPSC amplitudes were attained (15–25 min), before addition of ACPT-1. Fig. 7Aa shows voltage clamp recordings from one neurone illustrating a train of eEPSCs. A clear reduction in amplitude of all eEPSCs was seen following blockade of N-type VGCCs with CTx. However, subsequent application of ACPT-1 in the presence of CTx further reduced the amplitude of the eEPSCs. The plot in Fig. 7Ab shows the average responses across three trains in each of 17 neurones, in control conditions and in the presence of CTx, with and without the addition of ACPT-1. It is clear that the application of ACPT-1 still causes a substantial reduction in eEPSC amplitudes in the presence of the toxin. The bar chart in Fig. 6Ac illustrates the normalized fractional amplitude of eEPSCs in all neurones calculated across all 20 events in four trains for each neurone in each condition.

Similar results were obtained following inhibition of P/Q type VGCCs with AgTx, and a representative study is shown in Fig. 7Ba. Again, the raw data show the reduction in eEPSC amplitudes with AgTx and a subsequent further reduction with the introduction of ACPT-1. The plots in Fig. 7Bb were constructed from seven neurones, and clearly show that the toxin did not prevent the ability of ACPT-1 to reduce eEPSC amplitude in this cell. The summary data in Fig. 7Bc again illustrate averaged data across trains for the seven neurones.

Overall, AgTx and CTx had similar effects and this is reinforced by comparing the pooled data analysis from a number of studies in Fig. 7Ac and 7Bc. The normalized mean fractional amplitude of eEPSCs was reduced to 0.75±0.02 (*P*<0.01) in the presence of CTx, and to 0.76±0.04 by AgTx (*P*<0.01). Addition of ACPT-1 saw further reductions to 0.49±0.02 and 0.52±0.03, respectively (*P*<0.01 in both cases). What is clear is that ACPT plus either toxin results in a total reduction similar to that seen with ACPT alone (Fig. 6).

In a third set of experiments we investigated the possible contribution of R-type VGCCs to the effect of group III mGluRs. In the presence of the tarantula toxin, SNx (1 μM), which is thought to be specific for R-type channels (Newcomb et al., 1998; but see Arroyo et al., 2003), the ability of ACPT-1 to depress eEPSC amplitude was similar to that seen with ACPT alone. Fig. 7Ca illustrates one study. As with CTx and AgTx, SNX (*n*=6) reduced the amplitude of the eEPSCs, but this effect was weak, was largely confined to the early, facilitated responses in the train (Fig. 7Ca and 7Cb). Indeed, in pooled data averaging across the trains in all neurones the toxin appeared without overall effect (Fig. 7Cc). However, addition of ACPT-1 in the presence of SNx markedly reduced eEPSC amplitude (Fig. 7Ca and 7Cb). This effect is clearly demonstrated by the pooled data, particularly in comparison to the other toxins. Mean fractional eEPSC amplitude was unaffected by SNX (0.99±0.04), but with subsequent addition of ACPT-1 it decreased to 0.50±0.03 (*P*<0.01), representing a 50% decrease in amplitude compared with the toxin alone.

In a further set of experiments (Fig. 7D) we used Ni^2+^ (50 μM) to block R-type channels instead of SNx, and saw very similar results. In this case, the mean fractional amplitude in the presence of the divalent cation was 0.84±0.03, but in the presence of ACPT-1 the additional reduction to 0.32±0.01 appeared to be more pronounced (Fig. 7Db, c) *P*<0.01), reflecting a reduction by the agonist of approximately 62%. This could be construed as a potentiation of the effects of ACPT-1 by R-channel blockade.

Overall, it appears that N, and P/Q type channels contribute to the Ca^2+^-influx underlying glutamate release at these terminals, and to approximately the same extent (25–30%). The contribution of R-type channels to evoked release is difficult to assess definitively using current pharmacological tools, since SNx failed to significantly affect eEPSCs overall, while Ni^2+^ induce a weak depression. However, in some cells (e.g. Fig. 6Ca, b) SNx seemed to reduce eEPSC amplitudes early in the train, perhaps suggesting that R-type channels may contribute to the initial facilitation. In contrast Ni^2+^ depressed events throughout the train. It is possible that this could reflect a non-specificity, and that Ni^2+^ blocks not only R-type channels but may partially block either or both P/Q and N-type channels in our experiments. Whatever the contribution to release made by R-type channels, it is clear that inhibition of either P/Q or N-type Ca^2+^-channels alone is insufficient to prevent the full effects of mGluR activation on eEPSC amplitude.

**Fig. 8:**
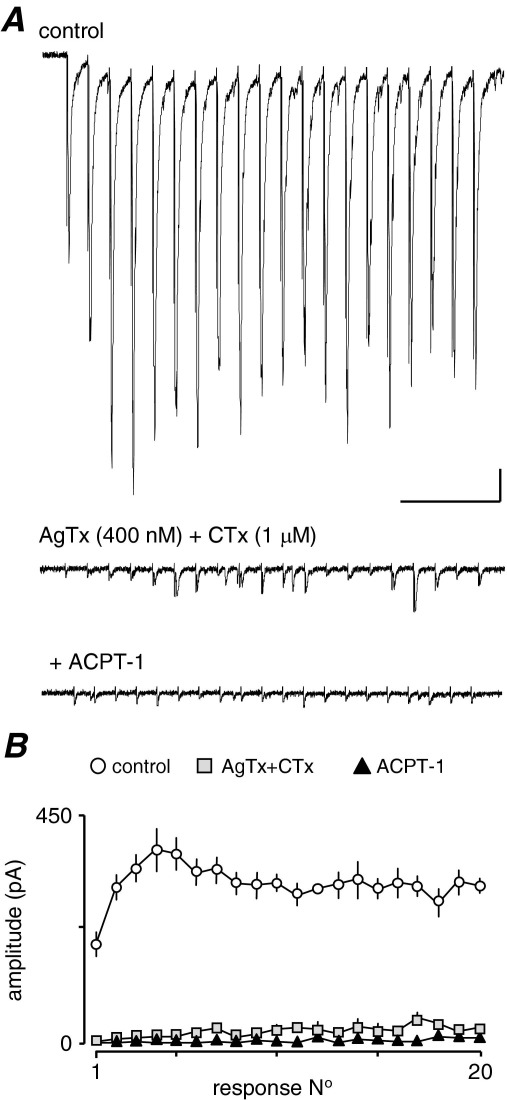
eEPSCs are dramatically reduced following combined application of CTx and AgTx. (A) The two toxins applied together left only a few small eEPSCs, toward the end of the train. These were essentially eliminated by ACPT-1. In all other neurones (*n*=4), eEPSCs were abolished by the toxins. (B) Average plot for four trains each in control, toxins and toxins plus ACPT-1 from the neurone in A. Scale bar=25 pA, 250 ms.

Currently, the data suggest that the effects of the group III mGluRs may involve both P/Q and N-type channels but not R-type. We attempted to test this further by simultaneously blocking P/Q and N-type Ca^2+^-channel subtypes with CTx and AgTx. However, in all but one recording (*n*=5), the combination of toxins completely abolished the eEPSCs, showing that simultaneous inhibition of P/Q or N-type VGCCs reduced the intraterminal Ca^2+^-transient to such a degree that release could not be initiated in most cases. In the remaining neurone (Fig. 8), there were small residual eEPSCs in the train resistant to combined toxins. Interestingly, these were abolished by ACPT-1, but it is clear that the CTx/AgTx combination reduced eEPSC amplitudes by >95% (Fig. 8), so support for a contribution of R-type channels to release at these synapses is weak. Recent reports indicating that R-type Ca^2+^-channels are only weakly coupled to release, and that alone, they are insufficient to support release (e.g. Wu et al., 1998, 1999) support this interpretation. Thus, we conclude that N, P/Q primarily carry the Ca^2+^-influx underlying glutamate release at these terminals.

## Discussion

### Evoked and AP-independent glutamate release are differentially modulated by mGluRs

We have shown that activation of a group III mGluR depresses eEPSC amplitude concurrently with an increase in sEPSC frequency (Evans et al., 2000a, 2001), suggesting that AP-dependent and independent glutamate release may be differentially modulated by this receptor in layer V of the EC. It seems likely that these effects are mediated through mGluR4 and/or mGluR8 since, at the concentration we used, ACPT-1 has similar affinity for these two receptors (approximately 8 nM; De Colle et al., 2000) and a comparatively low affinity for mGluR7. Of these group III receptors, mRNAs for mGluRs 4 and 7 have been demonstrated in the EC, but there is little information in the literature concerning mGluR8. However, one study has suggested that mGluR8 may be restricted to the very superficial layers (Kinoshita et al., 1996), so it is possible that our effects in the deep layers may be mediated by mGluR4. Further anatomical and pharmacological studies will be needed to answer this question.

Whatever the receptor involved, it is likely that effects on evoked and monoquantal glutamate release are mediated through inhibition of presynaptic Ca^2+^-channels and activation of AC, respectively. The demonstration that AP-dependent and independent release can be modulated in divergent directions is unusual but not unique. Activation of the VR1-receptor on primary afferent terminals in the dorsal horn increases mEPSC frequency while depressing eEPSCs (Baccei et al., 2003), and GABA_A_-receptors increase spontaneous release of glycine but decrease evoked release onto commissural nucleus neurones (Jang et al., 2002). Kondo and Marty (1997, 1998) demonstrated that noradrenaline increased the frequency and amplitude of sIPSCs in cerebellar stellate neurones, whereas only the frequency of mIPSCs was increased.

We found that mEPSCs and sEPSCs are increased in frequency by ACPT-1 (Evans et al., 2000a, 2001), but that stimulus-evoked eEPSCs were depressed by mGluR activation. If we assume that the pool of vesicles available for evoked release is the same as that for mEPSCs, then an increase in mEPSCs by mGluR activation could deplete the releasable pool available for the eEPSC. A number of studies have suggested that the size of the readily releasable pool of vesicles (RRP) correlates with the probability of release, such that an increased probability of release should also increase the size of the RRP (Rosenmund and Stevens, 1996; Dobrunz and Stevens, 1997; Goussakov et al., 2000). However, we have shown that activation of PKA increases both mEPSC frequency (Evans et al., 2001) and eEPSC amplitude indicating that the depression of evoked release by ACPT-1 may not be due to depletion of the RRP subsequent to increased spontaneous release. The relationship between RRP and release may be complex. For example, recent studies of group III mGluR in the medial nucleus of the trapezoid body (Billups et al., 2005) strongly suggest that the main action of mGluRs is to depress release probability (P), but this is matched by an increase in the size of the RRP of glutamate vesicles (N). In this scenario, mGluRs act not to suppress evoked release; rather, they enable redistribution of metabolic demand at the terminal. Certainly, it is plausible that as P declines and N is reciprocally enhanced, eEPSC depression is a sequel to reduced P, and enhanced spontaneous release reflects a larger RRP. It seems likely that eEPSC depression follows Ca^2+^ channel inhibition, which directly decreases release probability through reduction in the intra-terminal Ca^2+^ signal. It is also clear that the cAMP signaling machinery has the ability to mediate enhancement of the size of the RRP. Recent studies in drosophila terminals (see Kidokoro et al., 2004 for review) have indicated that mGluR-mediated alterations in cAMP initiate the shift of vesicles from the reserve pool to the RRP.

It is unlikely that PKA activation underlies the mGluR depression of eEPSCs at layer V synapses, since eEPSC amplitude was enhanced by forskolin, and the depression induced by ACPT-1 persisted in the presence of an AC inhibitor. Studies at other synapses support this facilitatory role for PKA activation in both spontaneous and evoked transmitter release (Chavez-Noriega and Stevens, 1994; Capogna et al., 1995; Sciancalepore et al., 1995; Chen and Regehr, 1997; Kondo and Marty, 1997). Interestingly, Cai et al. (2001) have shown that PKA may reduce mGluR function by phosphorylation of a serine residue in the C-terminal tail of the receptor. The ability of mGluR to activate PKA, and for PKA to inhibit mGluRs raises very interesting possibilities with respect to feedback regulation of glutamate release.

### Presynaptic Ca^2+^-channels and depression of glutamate release

We found that eEPSCs in layer V neurones were reduced in amplitude following blockade of either P/Q or N-Type channels. SNx or Ni^2+^, which show some selectivity for block of R-type channels (see Tottene et al., 1996; Wu et al., 1998, but also Arroyo et al., 2003), also reduced release. However, SNx only affected the facilitation of release occurring early during repetitive stimulation. Ni^2+^ weakly depressed release throughout the period of stimulation. Thus, it is possible that R-type channels may contribute to frequency facilitation of eEPSCs, and that the effects of Ni^2+^ are due to non-specific blockade of R, P/Q and N-type channels. Since, concurrent application of both AgTx and CTx virtually eliminated eEPSCs, we interpret this to mean that R-type channels alone are probably insufficient to support transmitter release at excitatory synapses in layer V. This would agree with previous conclusions that R-type channels control release much less effectively than P/Q or N-channels (Wu et al., 1998, 1999).

Inhibition of either P/Q or N-type Ca^2+^-channels alone was insufficient to prevent the full effects of mGluR activation. It seems likely that when P/Q-channels were blocked with AgTx, N-type channels would still be partly depressed by ACPT-1, sufficient to mediate the full effect of mGluR activation, and vice versa when N-type channels were blocked with CTx. Specific blockade of R-type channels with SNx did not have any overall effect on the ability of group III mGluR activation to depress eEPSCs. However, Ni^2+^ actually seemed to enhance the depression of eEPSCs by ACPT-1. A likely explanation of this is that the divalent cation was already weakly blocking P/Q and/or N-type channels, and compounding the additional reduction of channel activity due to group III receptor activation. Millan et al. (2002), have demonstrated that subtypes of group III mGluRs are associated with specific patterns of Ca^2+^-channel expression in glutamate terminals. mGluR4 is associated with terminals bearing both N and P/Q-type channels and can inhibit both, while mGluR7 is expressed at terminals bearing predominantly N-type channels (Millan et al., 2002). On the other hand, Takahashi et al. (1996) have suggested that the group III agonist, L-AP4, specifically targets P/Q channels to reduce activity-dependent glutamate release, but Wu et al. (1998) have also shown that it can partially inhibit R-type channels at the same synapses. Our data suggest that ACPT-1 has little effect on R-type channels, but we have not been able to examine its effect on synaptic responses supported purely by R-type channels.

We cannot rule out the possibility that ACPT-1 may depress evoked release by interacting with the release process downstream of Ca^2+^-influx. Other studies have suggested that group III (and possibly other) mGluRs can depress glutamate and GABA release independently of Ca^2+^-channel inhibition (Gereau and Conn, 1994; Llano and Marty, 1995; Scanziani et al., 1995; Schoppa and Westbrook, 1997; Krieger et al., 1999). Interestingly, Rusakov et al. (2004) have shown that L-AP4 reduces eIPSCs, depresses AP-dependent Ca^2+^-transients in the presynaptic GABAergic terminals, and occludes the effects of Ca^2+^-channel blockers in these processes, but the authors still acknowledge that downstream effects of the agonist could contribute to inhibition of GABA release.

The molecular mechanism underlying the effect of mGluR4 on eEPSCs is a matter for speculation. Assuming that it involves modulation of N and/or P/Q-type channels, then a likely explanation is via direct interaction of the Gβγ-subunit with the Ca^2+^-channel (see Jarvis and Zamponi, 2001; Dolphin, 2003). O’Connor et al. (1999) have suggested that release of Gβγ subunits from the carboxy-terminal tail of group III mGluRs requires activation of calmodulin, and it is possible that such a mechanism may be involved in the effect of mGluRs. However, Gβγ-subunits have also been suggested to inhibit glutamate release via downstream effects on the exocytotic machinery (Blackmer et al., 2001). There is also evidence that mGluRs may exert G-protein-independent effects on ion channels and transmitter release (see Heuss and Gerber, 2000).

### Differential actions of mGluR via separate signaling pathways

It is clear that mGluRs have divergent effects on AP-dependent and independent release of glutamate, suggesting that a single receptor subtype can couple to more than one effector system. A number of G-protein-coupled receptors have divergent effects on K^+^-channels and Ca^2+^-channels (e.g. Lledo et al., 1992; Wilk-Blaszczak et al., 1994; Vaughan et al., 2001). mGluR1 inhibits Ca^2+^-channels via multiple transduction pathways in HEK 293 cells (McCool et al., 1998) and in CA3 pyramidal cells the same receptor simultaneously elicits a G-protein-dependent slow AHP, and a G-protein-independent slow depolarization via activation of a tyrosine kinase (Heuss et al., 1999). It has often been presumed that spontaneous and evoked releases arise via the same exocytotic mechanisms, but rely on Ca^2+^ from different sources (intracellular stores versus influx via VGCCs). However, recent studies have suggested that the proteins involved in vesicle docking and fusion may be different for the two forms of release (Deitcher et al., 1998; Hua et al., 1998), and that the role of phosphorylation in regulation of glutamate release differs for spontaneous and evoked events (Oleskevich and Walmsley, 2000; Waters and Smith, 2000). Similarly, studies in drosophila (Kidokoro et al., 2004) have shown that while the RRP and the reserve pool exchange vesicles, they are replenished separately. Therefore, while vesicles for the two forms of release may be derived from the same RRP, this does not necessarily mean that the two processes must be regulated in parallel, and it is not unreasonable to suggest that group III mGluRs mediate a differential effect on release via two separate signaling pathways.

### Functional consequences of mGluR activation

The current experiments agree with previous observations that group III mGluRs can depress activity-dependent excitation at cortical synapses. The fact that blockade of the receptors enhanced the early responses (including the first) during repetitive stimulation indicates that they are constitutively activated to dampen glutamate release at low frequency, and to limit frequency facilitation. The functional role of the enhancement of activity-independent release by group III receptors is less obvious. We have previously shown that this glutamate-induced glutamate release does seem to be functionally active in the EC. Thus, neurokinin receptor agonists dramatically increase sEPSC frequency in layer V neurones, and this effect is partially ameliorated by a group III antagonist (Stacey et al., 2002), indicating that modest excitation of mEC is sufficient to elicit enhanced glutamate release. Interestingly, when sEPSCs are enhanced in this way, eEPSCs are concurrently depressed (A. Stacey and R. S. G. Jones, unpublished observations), so there may be a complex functional interrelationship between the mechanistically separable effects of group III mGluRs on activity-dependent and -independent release. We have also shown that presynaptic NMDA receptors in mEC tonically facilitate spontaneous glutamate release (Berretta and Jones, 1996b; Woodhall et al., 2001b). Interestingly, the positive-feedback effects on glutamate release by both NMDA receptors and mGluRs are developmentally regulated, such that they are greatly diminished at 6 months, compared with 1–3 months when most of our studies, including those in the current investigation, are conducted (NMDA, Yang et al., 2006; mGluR, G. L. Woodhall and R. S. G. Jones, unpublished observations). Thus the inherently unstable positive feedback of glutamate on glutamate release may be largely restricted the developing EC, perhaps reflecting processes involved in activity-dependent synaptogenesis, maintenance and elimination.
